# PPDPF Promotes the Development of Mutant KRAS‐Driven Pancreatic Ductal Adenocarcinoma by Regulating the GEF Activity of SOS1

**DOI:** 10.1002/advs.202202448

**Published:** 2022-12-01

**Authors:** Qian‐Zhi Ni, Bing Zhu, Yan Ji, Qian‐Wen Zheng, Xin Liang, Ning Ma, Hao Jiang, Feng‐Kun Zhang, Yu‐Rong Shang, Yi‐Kang Wang, Sheng Xu, Er‐Bin Zhang, Yan‐Mei Yuan, Tian‐Wei Chen, Fen‐Fen Yin, Hui‐Jun Cao, Jing‐Yi Huang, Ji Xia, Xu‐Fen Ding, Xiao‐Song Qiu, Kai Ding, Chao Song, Wen‐Tao Zhou, Meng Wu, Kang Wang, Rui Lui, Qiu Lin, Wei Chen, Zhi‐Gang Li, Shu‐Qun Cheng, Xiao‐Fan Wang, Dong Xie, Jing‐Jing Li

**Affiliations:** ^1^ CAS Key Laboratory of Nutrition Metabolism and Food Safety Shanghai Institute of Nutrition and Health University of Chinese Academy of Sciences Chinese Academy of Sciences Shanghai 200031 P. R. China; ^2^ Department of Hepatic Surgery VI Eastern Hepatobiliary Surgery Hospital Naval Medical University Shanghai 200433 P. R. China; ^3^ School of Life Science and Technology ShanghaiTech University Shanghai 201210 P. R. China; ^4^ Department of Thoracic Surgery Section of Esophageal Surgery Shanghai Chest Hospital Shanghai Jiao Tong University Shanghai 200030 P. R. China; ^5^ Department of Biomedical Informatics School of Life Sciences Central South University Changsha 410013 P. R. China; ^6^ Department of Pancreatic Surgery Zhongshan Hospital Fudan University Shanghai 200032 P. R. China; ^7^ Center for Excellence in Molecular Cell Science Chinese Academy of Sciences Shanghai 200031 P. R. China; ^8^ State Key Laboratory of Genetic Engineering School of Life Sciences Fudan University Shanghai 200438 P. R. China; ^9^ Cancer Institute of Integrated Traditional Chinese and Western Medicine Tongde Hospital of Zhejiang Province Hangzhou Zhejiang 310012 P. R. China; ^10^ Department of Pharmacology and Cancer Biology Duke University Medical Center Durham NC 27710 USA; ^11^ NHC Key Laboratory of Food Safety Risk Assessment China National Center for Food Safety Risk Assessment Beijing 100022 P. R. China

**Keywords:** KRAS, pancreatic ductal adenocarcinoma, pancreatic progenitor cell differentiation, proliferation factor, SOS1

## Abstract

The guanine nucleotide exchange factor (GEF) SOS1 catalyzes the exchange of GDP for GTP on RAS. However, regulation of the GEF activity remains elusive. Here, the authors report that PPDPF functions as an important regulator of SOS1. The expression of PPDPF is significantly increased in pancreatic ductal adenocarcinoma (PDAC), associated with poor prognosis and recurrence of PDAC patients. Overexpression of PPDPF promotes PDAC cell growth in vitro and in vivo, while PPDPF knockout exerts opposite effects. Pancreatic‐specific deletion of PPDPF profoundly inhibits tumor development in KRAS^G12D^‐driven genetic mouse models of PDAC. PPDPF can bind GTP and transfer GTP to SOS1. Mutations of the GTP‐binding sites severely impair the tumor‐promoting effect of PPDPF. Consistently, mutations of the critical amino acids mediating SOS1–PPDPF interaction significantly impair the GEF activity of SOS1. Therefore, this study demonstrates a novel model of KRAS activation via PPDPF‐SOS1 axis, and provides a promising therapeutic target for PDAC.

## Graphical abstract

This study reveals a novel model of KRAS activation via PPDPF‐SOS1 axis. PPDPF is upregulated in pancreatic ductal adenocarcinoma. It can bind GTP and offer GTP to SOS1, which stimulates the GEF activity of SOS1 and subsequent activation of KRAS. This work provides a promising therapeutic target for PDAC.

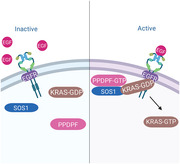

## 1 Introduction

Pancreatic ductal adenocarcinoma (PDAC) is the sixth most common cause of death from cancer worldwide. It was estimated that 495 773 new cases were diagnosed and 466 003 patients succumbed to this malignancy in 2020.^[^ ^1^ ^]^ The overall 5‐year survival rate for pancreatic cancer is less than 5%,^[^ ^2^ ^]^ and pancreatic cancer is projected to surpass breast, prostate and colorectal cancers to become the second leading cause of cancer‐related death by 2030.^[^ ^3^ ^]^ Although surgery is the only treatment that offers the prospect of long‐term survival for PDAC patients, the 5‐year survival rate for patients in whom tumor resection is possible remains less than 25%.^[^ ^4^ ^]^ In the past decade, despite a substantial increase in the understanding of pancreatic cancer, the therapeutic options remain limited.^[^ ^4b^ ^]^ Therefore, exploring the molecular mechanism underlying the tumorigenesis of PDAC will improve the understanding of the pathogenesis of PDAC and provide potential molecular targets for PDAC treatment.

RAS gene is the most frequently mutated oncogene in human cancers.^[^ ^5^ ^]^ KRAS mutations occur in more than 90% of PDACs.^[^ ^6^ ^]^ The predominant substitution is G12D, followed by G12V and G12R, whereas G13 and Q61 mutations are rare in pancreatic cancer.^[^ ^6^ ^]^ RAS is converted from an inactive GDP‐bound state to an active GTP‐bound state by RAS guanine nucleotide exchange factors (GEFs).^[^ ^7^ ^]^ RAS GEF, such as SOS1 is recruited to plasma membrane and catalyzes the exchange of GDP for GTP on RAS to turn on the signaling upon EGFR activation.^[^ ^7^ ^]^ The core domains of SOS1, CDC25 and REM domains (together named Cat domain), provide the catalytic activity toward RAS.^[^ ^8^ ^]^ The REM domain contains an activating allosteric site binding RAS‐GTP, which leads to additional stimulation of the catalytic CDC25 domain, and potentiates GDP–GTP exchange.^[^ ^8^ ^]^ The GEF activity of SOS1 is controlled by different mechanisms. The N‐terminal segment of SOS1 contains two tandem histone folds (the histone domain), followed by Dbl homology (DH) and the pleckstrin homology (PH) domains,^[^ ^8^ ^]^ which are essential for membrane recruitment^[^ ^9^ ^]^ and autoinhibition of SOS1.^[^ ^10^ ^]^ The serine phosphorylation of SOS1 in its C‐terminal domain by MAPK was reported to alter its association with Grb2 and inhibit the GEF function.^[^ ^11^ ^]^ A previous study reported that GEF could bind GTP.^[^ ^12^ ^]^ However, little is known about the significance of GTP‐binding for GEF and whether SOS1 could bind GTP.

Pancreatic progenitor cell differentiation and proliferation factor (PPDPF) was first reported in zebrafish.^[^ ^13^ ^]^ PPDPF is a key regulator of exocrine pancreas development, which has potential PDZ, SH2, SH3 domain binding sites and a GTP‐binding site.^[^ ^13^ ^]^ Recently, it was reported that circular RNA circ‐FOXM1 facilitated cell progression as ceRNA to target PPDPF and MACC1 by sponging miR‐1304‐5p in non‐small cell lung cancer,^[^ ^14^ ^]^ indicating the involvement of PPDPF in lung cancer. Another study reported that the prognosis of HCC patients with high expression of PPDPF was poor.^[^ ^15^ ^]^ However, the biological function of PPDPF in pancreatic cancer remains unknown.

The current study revealed that the expression of PPDPF was increased in pancreatic cancer, and patients with high PPDPF expression had a worse prognosis. Knockout of *Ppdpf* significantly inhibited tumor development in the mouse models of mutant KRAS‐driven PDAC. Moreover, we demonstrated that PPDPF could bind GTP, and offer GTP to SOS1, which stimulated its GEF activity and subsequent activation of KRAS. In conclusion, our study has revealed the novel function and underlying mechanisms of PPDPF in PDAC, providing a promising therapeutic target for this challenging malignancy.

## 2 Results

### 2.1 The Expression Pattern and Clinical Significance of PPDPF in PDAC

**Figure 1:**
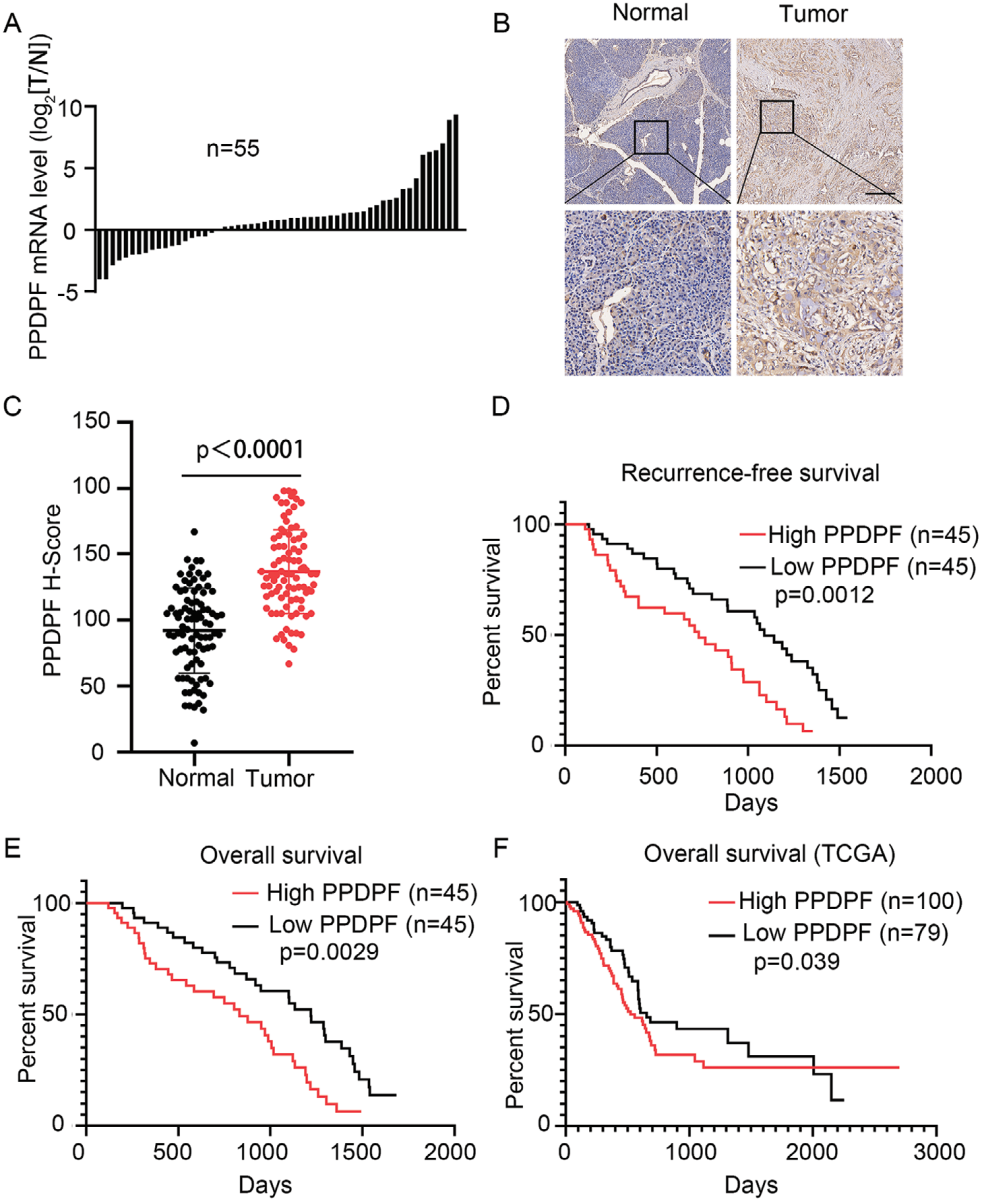
The expression pattern and clinical significance of PPDPF in PDAC. A) PPDPF mRNA levels in 55 pairs of tumor samples (T) and the matched adjacent noncancerous tissues (N) were examined by RT‐qPCR. B) Representative immunohistochemical staining of PPDPF in paired N and T tissues from PDAC patients, Scale bar, 100 µm. C) H‐scores of PPDPF expression in 90 pairs of PDAC tissues and the matched adjacent noncancerous tissues, and the median value was 135 (PPDPF high, high score ≥ 135, *n* = 45; PPDPF low, high score < 135, *n* = 45; *p* < 0.0001). D,E) The Recurrence‐free survival (PPDPF high, *n* = 45; PPDPF low, *n* = 45; *p* = 0.0012) and the overall survival curve (PPDPF high, *n* = 45; PPDPF low, *n* = 45; *p* = 0.0029) of PDAC patients with high or low PPDPF expression. F) The overall survival curve (PPDPF high, mRNA expression ≥ 4986, *n* = 100; PPDPF low, mRNA expression < 4986, *n* = 79; *p* = 0.039) of pancreatic cancer patients with high or low PPDPF expression from TCGA data. Survival curves were plotted using the Kaplan–Meier method and analyzed by the log‐rank test. Other data were analyzed by two‐tailed unpaired Student's *t* test. Data were expressed as mean ± SD.

To determine the expression pattern of PPDPF in PDAC, we used RT‐qPCR to examine the mRNA level of PPDPF in PDAC tissues and the matched adjacent noncancerous tissues. PPDPF mRNA levels were upregulated in 67.3% (37/55) of PDAC tissues compared with their counterpart (**Figure** **1**A). To further confirm the expression pattern of PPDPF in PDAC, the tissue microarray was stained with antibody against PPDPF. Consistent with results of RT‐qPCR, PPDPF expression was significantly increased in the tumor tissues compared to the paired noncancerous tissues (Figure 1B,C). The analysis of the relationship between PPDPF expression and the survival of PDAC patients revealed that high PPDPF expression was associated with poor recurrence‐free survival and overall survival (Figure 1D,E), which was similar to the TCGA data (Figure 1F).

According to the expression level of PPDPF, PDAC patients were divided into two groups: PPDPF low (H‐score < 135) and PPDPF high (H‐score ≥ 135). The relationship between PPDPF expression and the clinicopathological features of 90 PDAC patients were analyzed. As shown in Table S1, Supporting Information, PPDPF expression level was positively associated with tumor size (*p* = 0.031), TNM Stage (*p* = 0.018), and histological grade (*p* = 0.038). These data suggested that PPDPF could serve as a biomarker for PDAC and may have a tumor‐promoting role in PDAC.

### 2.2 PPDPF Promotes the Growth and Tumorigenesis of PDAC Cells

**Figure 2:**
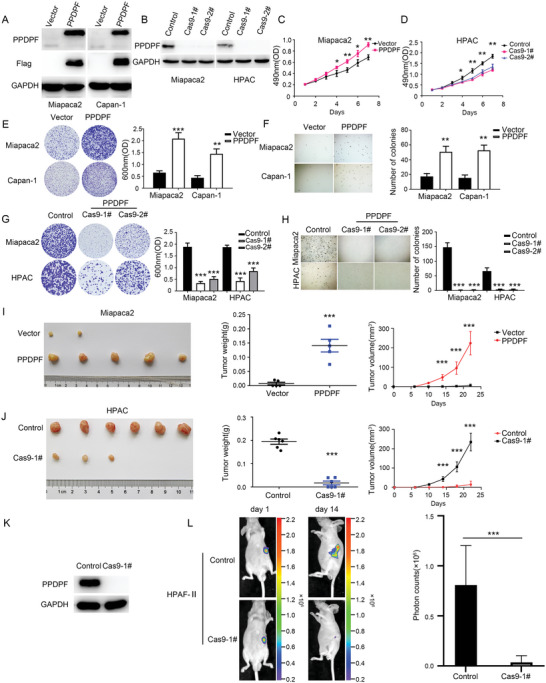
PPDPF promotes the growth and tumorigenesis of PDAC cells. A,B) The protein levels of PPDPF in control, PPDPF‐overexpressing and PPDPF knockout PDAC cells were examined by western blotting. C,D) The effects of PPDPF overexpression (C) or knockout (D) on the viability of pancreatic cancer cells were evaluated by MTT assay. E,F) Crystal violet assay and soft agar assay were used to determine the colony formation ability (E) and anchorage‐independent growth (F) of control and PPDPF‐overexpressing PDAC cells. G,H) Crystal violet assay and soft agar assay were used to determine the colony formation ability (G) and anchorage‐independent growth (H) of control and PPDPF knockout PDAC cells. I,J) Images, growth curves and weights of the tumors generated by control, PPDPF‐overexpressing Miapaca2 cells (*n* = 5) or PPDPF knockout HPAC cells (*n* = 6). K) The protein levels of PPDPF in control and PPDPF knockout HPAF‐II cells were examined by western blotting. L) Bioluminescent images of the mice intrapancreatically injected with control or PPDPF knockout HPAF II cells monitored at day 1 and day 14 (left). The photon counts were measured at day 14 (right, *n* = 6, *p* = 0.0008). Data were analyzed by two‐tailed unpaired Student's *t* test. Data were expressed as mean ± SD. *p < 0.05; **p < 0.01;***p < 0.001.

The clinical data suggested that PPDPF may promote the growth of PDAC cells. Thus, we constructed PPDPF‐overexpressing Miapaca2 and Capan‐1 cells, as well as PPDPF knockout Miapaca2 and HPAC cells to explore its function. The efficiency of PPDPF overexpression and depletion in PDAC cells was validated by western blotting (**Figure** **2**A,B). As expected, PPDPF overexpression significantly increased the growth of PDAC cells in vitro, which was revealed by MTT (Figure 2C and Figure S1A, Supporting Information), colony formation (Figure 2E) and soft agar assays (Figure 2F). Consistently, PPDPF knockout remarkably decreased the proliferation of PDAC cells in vitro, which was detected by the same assays (Figures 2D,G,H, and Figure S1B, Supporting Information).

To identify the growth‐promoting function of PPDPF in vivo, vector control and PPDPF‐overexpressing Miapaca2 cells were injected into the flanks of nude mice, respectively, and tumor growth was monitored (Figure 2I). Tumors generated by PPDPF‐overexpressing Miapaca2 cells grew faster and were bigger than those derived from control cells (Figure 2I). In contrast, tumors generated by PPDPF knockout HPAC cells grew slower and were smaller than those derived from control cells (Figure 2J).

The tumor‐promoting effect of PPDPF was further validated in orthotopic PDAC mouse model. Luciferase‐labeled PPDPF knockout HPAF‐II cells and control cells were injected into the pancreas of nude mice, respectively. Two weeks after tumor cell implantation, the amount of photons in the PPDPF knockout group was much lower than that in the control group, despite the equal bioluminescent intensity at the beginning (Figure 2K). These data together suggested that PPDPF could promote the in vitro growth and in vivo tumorigenesis of PDAC cells, which was consistent with the clinical data.

### 2.3 Loss of PPDPF Profoundly Inhibits KRAS^G12D^‐Driven Pancreatic Carcinogenesis

**Figure 3:**
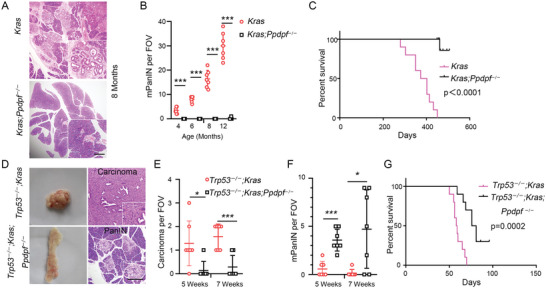
Loss of *Ppdpf* profoundly inhibits Kras^G12D^‐driven pancreatic carcinogenesis. A) Representative HE stained pancreatic sections from *Kras* and *Kras;Ppdpf^−/−^* mice at 8 months of age, Scale bar, 200 µm. B) Number of mouse PanINs (mPanINs) in *Kras* and *Kras;Ppdpf^−/−^* mice. mPanINs were quantified over the whole HE stained pancreatic section from mice at different ages (4 months, 6 months, 8 months and 12 months, *n* = 7 for each time point, *p* < 0.0001), FOV, field of view. C) Overall survival curve of *Kras* mice (*n* = 10, median survival time: 373 days) and *Kras;Ppdpf* ^−/−^mice (*n* = 10, median survival time was not reached, *p* < 0.0001). D) Representative images and HE stained sections of pancreas from *Trp53^−/−^*; *Kras* (*n* = 7) and *Trp53^−/−^*; *Kras; Ppdpf^−/−^* mice (*n* = 7) at 5 weeks of age, Scale bar, 100 µm. E,F) Number of carcinoma per FOV (E) and mPanIN per FOV (F) in *Trp53^−/−^; Kras* or *Trp53^−/−^; Kras; Ppdpf^−/−^* mice at the indicated age (5 weeks and 7 weeks, *n* = 7 for each group). G) Overall survival curve of *Trp53^−/−^; Kras* mice (*n* = 10, median survival time: 58 days) and *Trp53^−/−^*; *Kras; Ppdpf^−/−^* mice (*n* = 10, median survival time: 76 days, *p* = 0.0002). Survival curves were plotted using the Kaplan–Meier method and analyzed by the log‐rank test. Other data were analyzed by two‐tailed unpaired Student's *t* test. Data were expressed as mean ± SD. *p < 0.05; ***p < 0.001.

To dissect the contribution of PPDPF to PDAC tumorigenesis, oncogenic KRAS‐driven genetic mouse models of PDAC were employed.^[^ ^16^ ^]^ Pancreas‐specific deletion of *Ppdpf* in *Kras^G12D^* mice (termed *Kras*) profoundly inhibited the development of pancreatic intraepithelial neoplasia (PanIN) (**Figure** **3**A,B). *Ppdpf* deletion dramatically prolonged the survival of Pdx1‐Cre; *Kras* mice (Figure 3C). To further clarify the function of PPDPF in PDAC, the more aggressive and tumor‐prone PDAC mouse model with biallelic deletion of Trp53^[^ ^17^ ^]^ was employed. In this model, *Ppdpf* deficiency resulted in increased number of PanIN while decreased number of PDAC, indicating *Ppdpf* deletion also hindered the progression from PanIN to PDAC (Figure 3D–F). Furthermore, *Ppdpf* deficiency significantly extended the survival of mice (Figure 3G). Taken together, these data indicated a central and indispensable role of PPDPF in the development of KRAS^G12D^‐driven PDAC.

### 2.4 PPDPF Activates RAS/MPAPK Signaling in PDAC

**Figure 4:**
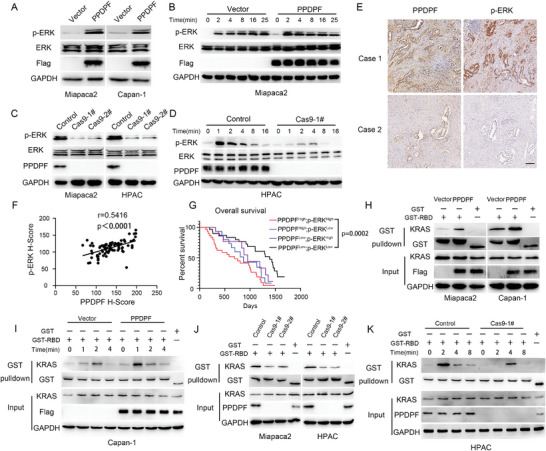
PPDPF activates RAS/MAPK signaling in PDAC. A) The expression of the indicated molecules in control and PPDPF‐overexpressing cells were examined by western blotting. GAPDH was used as internal control. B) Western blot analysis of p‐ERK in control and PPDPF‐overexpressing cells with EGF treatment. C,D) Western blot analysis of p‐ERK in control and PPDPF knockout cells without (C) or with (D) EGF treatment. E) Immunohistochemical staining of PPDPF and p‐ERK in PDAC tissues; Scale bar, 100 µm. F) Positive correlation between PPDPF and p‐ERK according to the H‐scores in PDAC tissues (*r* = 0.5416, *p* ＜ 0.0001). G) Overall survival curve of PDAC patients based on the expression levels of PPDPF and p‐ERK (PPDPF high, p‐ERK high, *n* = 30; PPDPF low, p‐ERK high, *n* = 15; PPDPF high, p‐ERK low, *n* = 15; PPDPF low, p‐ERK low, *n* = 30, *p* = 0.0002). H,I) the level of KRAS‐GTP in control and PPDPF‐overexpressing cells was detected by GST‐RBD pulldown assay without (H) or with (I) EGF treatment. J,K) the level of KRAS‐GTP in control and PPDPF knockout PDAC cells was examined by GST‐RBD pulldown assay without (J) or with (K) EGF treatment. Survival curves were plotted using the Kaplan–Meier method and analyzed by the log‐rank test. Correlation was analyzed by Pearson correlation analysis. Data were expressed as mean ± SD.

Considering the central role of RAS/MAPK signaling in the tumorigenesis of PDAC,^[^ ^18^ ^]^ we examined the influence of PPDPF on this signaling. We found that overexpression of PPDPF significantly elevated the level of p‐ERK, a well‐known marker of RAS activation,^[^ ^19^ ^]^ in pancreatic cancer cells (**Figure** **4**A), while PPDPF knockout remarkably decreased the expression level of p‐ERK (Figure 4C). We also observed faster activation of ERK in PPDPF‐overexpressing cells compared with control cells upon EGF treatment (Figure 4B and Figure S2A, Supporting Information). Consistently, ERK activation was dramatically inhibited in PPDPF knockout cells in response to EGF stimulation (Figure 4D and Figure S2B–D, Supporting Information).

To further identify the relationship between PPDPF and ERK activation, the expression level of p‐ERK was examined by immunohistochemical staining in the tissue microarray, and higher level of p‐ERK was observed in PDAC tissues compared with the adjacent nontumor tissues (Figure S2E, Supporting Information). In addition, obvious p‐Erk staining was detected in the PanIN tissues in Pdx1‐Cre; *Kras^G12D^* mice, while its level was much lower in Pdx1‐Cre; *Kras^G12D^*; *Ppdpf* ^f/f^ mice (Figure S2F, Supporting Information). The positive correlation between PPDPF expression and the level of p‐ERK was further confirmed in the PDAC samples (Figure 4E,F). Moreover, high PPDPF and p‐ERK expression levels were associated with much poorer overall survival (Figure 4G) compared with the PPDPF^low^, p‐ERK^low^ group in 90 PDAC patients.

Since RAS functions upstream of ERK,^[^ ^20^ ^]^ then we examined the influence of PPDPF on the level of active GTP‐bound KRAS by GST‐Raf‐binding domain (RBD) pulldown assay. Overexpression of PPDPF significantly elevated the level of KRAS‐GTP (Figure 4H), while PPDPF knockout remarkably decreased the level of KRAS‐GTP in PDAC cells (Figure 4J). We also observed that the level of KRAS‐GTP increased faster in PPDPF‐overexpressing cells compared with control cells upon EGF stimulation (Figure 4I and Figure S2G, Supporting Information). Consistently, KRAS activation was significantly inhibited in PPDPF knockout cells upon EGF treatment (Figure 4K and Figure S2H–J, Supporting Information). In summary, these data indicated that PPDPF promoted the activation of RAS/MAPK cascade.

### 2.5 The Tumor‐Promoting Effect of PPDPF Depends on the GEF Activity of SOS1

**Figure 5:**
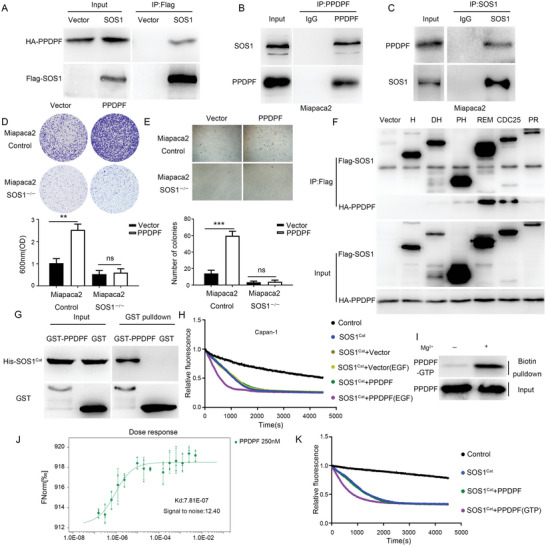
The tumor‐promoting effect of PPDPF depends on the GEF activity of SOS1. A) Interaction between exogenous PPDPF and SOS1 was detected by Co‐IP in 293T cells. B,C) Co‐IP performed with PPDPF (B) or SOS1 (C) antibody, respectively, in Miapaca2 cells. D,E) The growth of control and PPDPF‐overexpressing Miapca2 cells in the presence or absence of SOS1 was detected by Crystal violet assay (D) (*p* =0.0014) and soft agar assay (E) (*p* < 0.0001). F) The interactions between PPDPF and different domains of SOS1 were detected by Co‐IP in 293T cells. G) Interaction between PPDPF and SOS1^Cat^ was detected by GST pulldown assay in vitro. H) The GEF activity of SOS1^Cat^ with or without PPDPF/PPDPF (EGF) from Miapaca2 cells. I) GTP‐binding ability of PPDPF was examined by GTP binding assay. J) GTP‐binding ability of PPDPF was detected by MST assay, the signal was detected by Monolith NT.115 instrument. K) The GEF activity of SOS1^Cat^ with PPDPF or PPDPF‐GTP in vitro. Data were analyzed with two‐tailed unpaired Student's *t* test. Data were expressed as mean ± SD. **p < 0.01; ***p < 0.001, ns: not significant.

The above data indicated that PPDPF could elevate KRAS activity (Figure 4H,J). Considering the critical role of SOS1 in RAS activation,^[^ ^21^ ^]^ we checked whether PPDPF could influence SOS1 to activate KRAS. The interaction between PPDPF and SOS1 was first examined, and the Co‐IP assay in 293T cells disclosed the interaction between exogenous PPDPF and SOS1 (**Figure** **5**A). To verify this interaction, we tested the interaction between endogenous PPDPF and SOS1 in Miapaca2 cells, and obtained similar results (Figure 5B,C).

Next, we asked whether the tumor‐promoting effect of PPDPF in PDAC was SOS1‐dependent. We transfected SOS1 knockout Miapaca2 and HPAC cells with PPDPF expression construct or empty control vector, respectively. The results of MTT, colony formation and soft agar assays demonstrated that PPDPF overexpression significantly increased cell growth of SOS1 wildtype PDAC cells (Figure 5D,E and Figure S3A–D, Supporting Information), which was similar to Figure 2E,F. However, no significant alteration was observed in SOS1 knockout PDAC cells (Figure 5D,E and Figure S3A–D, Supporting Information). These data suggested that SOS1 was required for the growth‐promoting effect of PPDPF in PDAC cells.

To further clarify how PPDPF influence SOS1, their interaction was mapped by Co‐IP assay using different truncated mutants of SOS1. We found that PPDPF mainly interacted with the REM and CDC25 domain of SOS1 (Figure 5F), which constitute the catalytic module (named Cat domain).^[^ ^22b^ ^]^ Furthermore, the direct interaction between SOS1^Cat^ and PPDPF was further confirmed by in vitro GST pulldown assay (Figure 5G). Therefore, we supposed that PPDPF may influencethe GEF activity of SOS1. Flag‐tagged PPDPF protein from PDAC cells was incubated with SOS1^Cat^ and KRAS in GEF activity assay and only the PPDPF protein from EGF‐stimulated PDAC cells could promote the GEF activity of SOS1^Cat^ (Figure 5H and Figure S3E, Supporting Information), indicating that PPDPF may undergo some alteration upon EGF treatment. Domain prediction in the previous study suggested that PPDPF could bind GTP,^[^ ^13^ ^]^ which plays an important role in KRAS activation. Thus, we examined this possibility by GTP binding assay (Figure 5I) and verified it. Furthermore, the GTP‐binding capability of PPDPF was validated by MST assay (Figure 5J). Consistently, the GTP‐bound PPDPF recombinant protein could enhance the GEF activity of SOS1^Cat^, rather than PPDPF protein alone (Figure 5K). Taken together, these data suggested that GTP‐loaded PPDPF enhanced the GEF activity of SOS1, which was indispensable for the tumor‐promoting function of PPDPF in PDAC.

### 2.6 GTP‐Binding Ability Is Required for the Tumor‐Promoting Function of PPDPF

**Figure 6:**
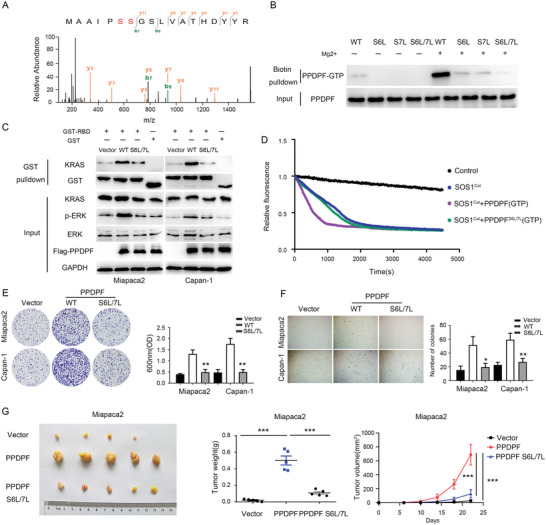
GTP‐binding ability is required for the tumor‐promoting function of PPDPF. A) The MS/MS spectrum of modified “MAAIPS^(GTP)^S^(GTP)^GSLVATHDYYR”. B) The GTP‐binding ability of WT PPDPF and the indicated mutants was detected by GTP binding assay. C) Western blot analysis of indicated molecules in control, PPDPF WT‐ and PPDPF(S6L/7L)–overexpressing PDAC cells. D) The GEF activity of SOS1^Cat^ with PPDPF‐GTP or PPDPF(S6L/7L)‐GTP in vitro. E,F) Crystal violet assay (E) (*p* = 0.0028 for Miapaca2, *p* = 0.0013 for Capan‐1) and soft agar assay (F) (*p* = 0.0138 for Miapaca2, *p* = 0.0035 for Capan‐1) were used to examine the colony formation ability and anchorage‐independent growth of the indicated PDAC cells. G) Images, growth curves and weights of the tumors generated by control, PPDPF WT‐ and PPDPF(S6L/7L)‐overexpressing Miapaca2 cells (*n* = 5, *p* < 0.001). Data were analyzed with two‐tailed unpaired Student's *t* test. Data were expressed as mean ± SD. *p < 0.05; **p < 0.01;***p < 0.001.

Mass spectrum (MS) was employed to find the GTP‐binding site within PPDPF, and Ser 6 and 7 was identified (**Figure** **6**A). Serines at these two sites were mutated to leucines according to the previous study,^[^ ^22^ ^]^ and three mutants were constructed (S6L, S7L, and S6L/7L). First, we tested the GTP‐binding capability of the mutants by GTP binding assay, and found that the GTP‐binding capability was severely impaired in all the three mutants, and PPDPF(S6L/7L) showed the lowest affinity to GTP (Figure 6B). To clarify their influence on RAS/MAPK signaling, we examined the level of p‐ERK and KRAS‐GTP in PDAC cells overexpressing PPDPF (WT) or PPDPF (S6L/7L). As shown in Figure 6C, the level of p‐ERK and KRAS‐GTP in PPDPF (S6L/7L)‐overexpressing PDAC cells was much lower compared with PPDPF (WT)‐overexpressing PDAC cells (Figure 6C). Consistently, PPDPF (S6L/7L) almost lost the ability to enhance the GEF activity of SOS1^Cat^ (Figure 6D). Moreover, the growth‐promoting effect of PPDPF was impaired by the S6L/7L mutation in vitro (Figure 6E,F and Figure S4A,B, Supporting Information) and in vivo (Figure 6G). In summary, the GTP‐binding capability of PPDPF was required for its tumor‐promoting effect via RAS/MAPK signaling.

### 2.7 GTP Transfer from PPDPF to SOS1 Is Indispensable for the Tumor‐Promoting Effect of PPDPF‐SOS1 Axis

**Figure 7:**
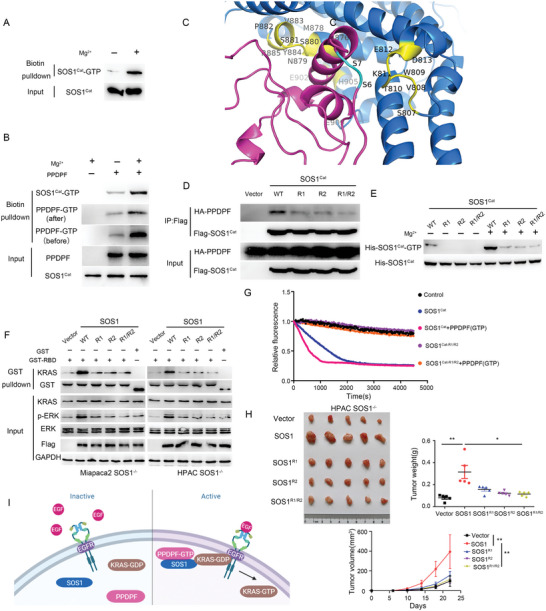
GTP transfer from PPDPF to SOS1 is indispensable for the tumor‐promoting effect of PPDPF‐SOS1 axis. A) GTP‐binding ability of SOS1^Cat^ was detected by GTP binding assay. B) The level of PPDPF‐GTP and SOS1^Cat^‐GTP was detected by GTP binding assay before and after incubation of SOS1^Cat^ with PPDPF‐GTP. C) The interacting sites at the interface between PPDPF and SOS1^Cat^ predicted by an integrated analysis. D) Interaction between PPDPF and SOS1^Cat^ and indicated SOS1^Cat^ mutants in 293T cells. E) The GTP‐binding ability of SOS1^Cat^ and the indicated SOS1^Cat^ mutants was examined by GTP binding assay. F) Western blot analysis of the indicated molecules in WT SOS1‐ and GTP‐binding‐defective SOS1 mutants‐overexpressing PDAC cells. G) The GEF activity of SOS1^Cat^ and SOS1^Cat‐R1/R2^ with or without PPDPF‐GTP. H) Images, weights (*p* = 0.0047 for SOS1 versus vector, *p* = 0.0101 for SOS1^R1/R2^ versus SOS1) and growth curves (*p* = 0.0061 for SOS1 versus vector, *p* = 0.0071 for SOS1^R1/R2^ versus SOS1) of the tumors generated by SOS1 knockout HPAC cells with reintroduction of SOS1, SOS1^R1^, SOS1^R2^ and SOS1 ^R1/R2^, respectively (*n* = 5 for each group). I) Schematic diagram of the model suggested by our study. Data were analyzed with two‐tailed unpaired Student's *t* test. Data were expressed as mean ± SD. *p < 0.05; **p < 0.01.

Previous study has indicated that GEF binds and utilizes GTP to complete the GDP–GTP exchange.^[^ ^12^ ^]^ Considering the GTP‐binding ability of PPDPF and its interaction with SOS1^Cat^ domain, we wondered whether GTP could be transferred from PPDPF to SOS1. As expected, SOS1 could bind GTP (**Figure** **7**A). To identify our hypothesis, SOS1^Cat^ was incubated with PPDPF‐GTP, and later, we found that the level of GTP‐bound PPDPF was decreased while SOS1^Cat^‐GTP increased, indicating that GTP was transferred from PPDPF to SOS1^Cat^ (Figure 7B). Next, we explored the mechanism underlying this GTP transfer. We utilized an integrated method to analyze the functional sites within SOS1^Cat^ mediating its interaction with PPDPF Ser6/7, which were supposed to be the GTP‐receiving sites. Based on the calculation,^[^ ^23^ ^]^ the amino acids showing strong interaction with Ser6/7 of PPDPF mainly reside in two regions, named Cat‐R1 and Cat‐R2 (Figure 7C and Figure S5A, Supporting Information). To clarify the function of the two regions, the critical amino acids were mutated into leucines, and three mutants SOS1^Cat‐R1^, SOS1^Cat‐R2^ and SOS1^Cat‐R1/R2^ were constructed, which represented the mutations within 807–814, 896–909 and 807–814/896–909 of SOS1^Cat^, respectively (the critical amino acids within the two regions are listed in Table S2, Supporting Information). The interaction between PPDPF and SOS1^Cat^ was profoundly weakened by the mutations (Figure 7D and Figure S5B, Supporting Information), which validated the prediction. Furthermore, all the three mutants exhibited remarkably reduced GTP‐binding ability (Figure 7E). The level of p‐ERK and KRAS‐GTP in SOS1^R1^, SOS1^R2^ and SOS1^R1/R2^ mutants‐overexpressing PDAC cells was much lower compared with SOS1 WT‐overexpressing PDAC cells (Figure 7F). Meanwhile, these mutants almost lost the GEF activity even in the presence of PPDPF‐GTP (Figure 7G and Figure S5C,D, Supporting Information).

It was worthy to note that the mutations did not influence the interaction between SOS1^Cat^ and KRAS (Figure S5E, Supporting Information). Thus, the interaction with PPDPF and GTP transfer from PPDPF directly regulated the GEF activity of SOS1, rather than influencing SOS1–KRAS interaction. Functionally, SOS1^R1^, SOS1^R2^ and SOS1^R1/R2^ had little effect on tumor cell growth in vitro (Figure S5F–I, Supporting Information) and in vivo (Figure 7H) in SOS1 knockout HPAC cells, while SOS1 WT showed a strong growth‐promoting effect. In conclusion, here we propose a novel model where PPDPF binds GTP and transfers it to SOS1 to enhance its GEF activity upon EGF stimulation, which subsequently activates KRAS and downstream oncogenic signaling (Figure 7I).

## 3 Discussion

PPDPF is a significant regulator of pancreatic exocrine cell specification and proliferation in zebrafish.^[^ ^13^ ^]^ Although several studies reported the role of PPDPF in cancer,^[^ ^15^ ^]^ its function in pancreatic cancer remains unclear. Our study reported the significantly increased PPDPF expression in PDAC, and found that its expression was associated with tumor size, TNM stage, histological grade, recurrence and survival, indicating it may serve as a prognostic marker for PDAC. Moreover, knockout of PPDPF significantly inhibited tumor development in the mouse models of KRAS^G12D^‐driven PDAC. These findings identified PPDPF as a novel and important oncoprotein in PDAC.

RAS GTPases cycle between GDP‐off and GTP‐on states.^[^ ^24^ ^]^ Mutation in RAS proteins is one of the most common genetic alterations in humans and is found in ≈30% of all cancers. Particularly, oncogenic KRAS mutants are observed in more than 90% pancreatic cancer.^[^ ^24^ ^]^ These mutants disable the GTP hydrolysis process and therefore impair its inactivation. Oncogenic RAS mutations are widely considered to be locked in a permanent “On” and “constitutively active” state. However, many healthy people have cells possessing mutant RAS without apparent harm, and mutant RAS causes transformation only after upregulation of RAS activity in animal models.^[^ ^24^ ^]^ Li et al. suggested that there is a narrow window or “sweet spot” by which oncogenic RAS signaling can promote tumor initiation in normal cells.^[^ ^7^ ^]^ Therefore, factors which could block RAS activation by upstream stimulants may have cancer‐preventive values in RAS mutant cancers. In our study, PPDPF knockout almost blocked the oncogenic activation of KRAS^G12D^ in mouse models, which is consistent with this opinion (Figure 3A,B,D,E). In addition, we found that PPDPF elevated the level of p‐ERK and KRAS‐GTP even in the absence of exogenous EGF (Figure 4A–H), while PPDPF knockout suppressed RAS/MAPK signaling in the resting state (Figure 4C–J). These findings suggest that PPDPF modulates the sensitivity of KRAS to EGF stimuli, which decreases the threshold for KRAS activation and enhances the oncogenic function of KRAS.

SOS1 is a key activator of the small GTPase RAS.^[^ ^25^ ^]^ Growth factor receptors activate RAS by recruiting SOS1 to cell membrane, which promotes the exchange of GDP for GTP on RAS, and triggers the production of GTP‐loaded RAS.^[^ ^7^ ^]^ SOS1 has two RAS binding sites: a catalytic site (CDC25 domain) and an allosteric RAS binding site (REM domain).^[^ ^26^ ^]^ Interestingly, we found that the two domains showed higher affinity to PPDPF than other domains (Figure 5F). The RAS GEF activity of SOS1 is delicately regulated, including membrane targeting,^[^ ^27^ ^]^ autoinhibition,^[^ ^28^ ^]^ allosteric alteration^[^ ^10^ ^]^ and serin/threonine phosphorylation of SOS1.^[^ ^11^ ^]^ Here our study provides an unreported regulation of SOS1. We found that PPDPF interacted with SOS1 (Figure 5A–C), both of them could bind GTP (Figures 5I and 7A) and PPDPF transferred GTP to SOS1 (Figure 7B). To our knowledge, this is the first report revealing GTP transfer between different proteins. We identified the Ser6,7 as the GTP‐binding sites within PPDPF by mass spectrum (Figure 6A), and an integrated method was employed to analyze the most possible amino acids within SOS1 mediating the interaction with PPDPF (Figure 7C), which were also supposed to be the GTP‐receiving sites. The importance of PPDPF–SOS1 interaction, and GTP transfer from PPDPF to SOS1 in KRAS activation and pancreatic cancer development was proved by the following evidence: first, the GTP‐binding defective mutants of PPDPF (S6L/7L) could not elevate KRAS‐GTP level (Figure 6C), and almost lost the growth‐promoting ability in vitro and in vivo (Figure 6E–G); second, interaction between PPDPF and SOS1 Cat mutants SOS1^Cat‐R1^/SOS1^Cat‐R2^/SOS1^Cat‐R1/R2^ was significantly decreased (Figure 7D and Figure S5B, Supporting Information); third, the GTP‐binding activity, KRAS GEF activity (Figure 7G and Figure S5C,D, Supporting Information) and tumor‐promoting function of SOS1 were severely impaired when either of the two regions was mutated (Figure 7H and Figure S5F,G, Supporting Information). Importantly, the two regions are in proximity to the KRAS binding site,^[^ ^29^ ^]^ but the mutations did not influence KRAS–SOS1 interaction (Figure S5E, Supporting Information). Therefore, our findings provide a comprehensive update on the current understanding of SOS1. However, it remains unclear how GTP affects SOS1 GEF activity, and protein structure study is required to address this issue.

In 1982, mutationally activated RAS genes were discovered in human cancers.^[^ ^30^ ^]^ Despite its well‐recognized importance in cancer malignancy, continuous efforts in the past three decades failed to develop approved therapies for KRAS‐mutant cancer. Just recently, KRAS G12C inhibitors have been approved by FDA.^[^ ^31^ ^]^ Nevertheless, 85% of KRAS‐mutated cancers still lack efficient therapeutic agents.^[^ ^30^ ^]^ The strategies to target KRAS include hindering KRAS membrane association,^[^ ^32^ ^]^ disrupting RAS dimerization/nanoclustering,^[^ ^33^ ^]^ interference with KRAS–SOS1 interaction,^[^ ^26^ ^]^ inhibition of the downstream effectors,^[^ ^32^ ^]^ synthetic lethality in KRAS mutant cancer,^[^ ^34^ ^]^ and so on. However, none of the efforts have been translated to clinical application, indicating the existence of blind points of KRAS signaling despite extensive studies. We may find one of the blind points. PPDPF knockout blocked the development of KRAS^G12D^‐driven PDAC in mouse models, and mechanistic study revealed that PPDPF regulated the GEF activity of SOS1 by providing it with GTP, which subsequently stimulated KRAS activation. Multiple features of PPDPF renders it a very attractive therapeutic target for PDAC, including the high expression in PDAC, the GTP‐binding ability, interaction with SOS1, and the capability to transfer GTP to SOS1.

In conclusion, the current study revealed the oncogenic role of PPDPF in PDAC by enhancing the GEF activity of SOS1 in a GTP‐dependent manner. Our findings not only reveal a novel regulation of SOS1, but also provide a promising therapeutic target for PDAC.

## 4 Experimental Section

### Cell Culture and Tumor Samples

Cells used in present study were gained from the Cell Bank of the Type Culture Collection of the Chinese Academy of Sciences. Capan‐1, HPAC, HPDE6C7, Miapaca‐2 and HEK293T cells were cultured in DMEM (Invitrogen) supplemented with 10% FBS (Anlite) and 10 U/mL penicillin G. HPAF‐II were cultured in MEM (Invitrogen) supplemented with 10% FBS (Anlite) and 10 U/mL penicillin G. All cells were incubated at 37 °C in a humidified atmosphere containing 5% CO2.^[^ ^35^ ^]^ The cell lines were tested to exclude mycoplasma contamination via PCR by GATC Biotech every 12 months, and for all the experiments, the cells were used within five passages after thawing.

After obtaining written informed consent, all PDAC and paired adjacent tissues were collected from Zhongshan Hospital, Fudan University. These experiments were approved by the Ethical Committee of institute for Nutritional Sciences, Chinese Academy of Sciences (Shanghai, China) following declaration of Helsinki ethical guidelines.

### Animals

Pdx1‐Cre, *Trp53* ^fl/fl^, and LSL‐*Kras^G12D^* mice were obtained from Jackson Laboratory and *Ppdpf* ^fl/fl^ was obtained from Nanjing University. Pdx1‐Cre; LSL‐*Kras^G12D^*; *Ppdpf* ^fl/fl^ was bred by crossing Pdx1‐Cre mice with LSL‐*Kras* ^G12D^; *Ppdpf^f^* ^l/fl^ mice. Pdx1‐Cre; LSL‐*Kras^G12D^*; P53^fl/fl^; *Ppdpf* ^fl/fl^ was generated by crossing Pdx1‐Cre with *Trp53* ^fl/fl^; LSL‐*Kras^G12D^*; *Ppdpf* ^fl/fl^. The sequences of primers used to identify the genotype of mice are listed in Table S3, Supporting Information. Six‐week‐old male BALB/c mice were housed under standard conditions. The animal protocols were complied with SIBS Guide for the Care and Use of Laboratory Animals and approved by Animal Care and Use Committee, Shanghai Institute of Nutrition and Health, Chinese Academy of Sciences.

### Tumorigenesis In Vivo

Suspended cells (5 × 10^5^) were injected into 6‐week‐old male nude mice treated in accordance with AAALAC criteria. Each animal was injected subcutaneously at two sites in their flanks. The tumor volume growth was monitored from the day of implantation. And tumor volume (mm^3^) was measured every four days and tumor weight was measured at the last.^[^ ^36^ ^]^

The pancreas orthotopic tumor implantation was performed as previously described.^[^ ^37^ ^]^ Orthotopic implantation was performed in 6‐week‐old male nude mice by first making a 5 to 10 mm transverse incision on the left flank of the mouse through the skin and peritoneum. The tail of the pancreas was then exposed through this incision. 2 × 10^6^ HPAF‐II cells were injected into the pancreas tail, which was subsequently returned into the abdomen. The incision was closed in two layers using 5.0 nonabsorbable sutures. The bioluminescence was monitored from the day of implantation.

### Cell Growth

Crystal violet and MTT assay performed as described previously were used to evaluate the proliferation ability of PDAC cells.^[^ ^38^ ^]^ The clonal ability of PDAC cells was detected by Soft Agar assay. The 24 well plate was moistened with deionized water after sterilization. 4 mL 2 × DMEM, 4 mL 1.25% agarose and 2 ml FBS were mixed and the mixed liquid was added into the 24 well plate (1 mL/well) as the lower part. In the meanwhile, 1 × 10^4^/mL tumor cells were counted and the cells were diluted with DMEM. Then 1 mL cells, 1 mL FBS, 1.5 mL 1% agarose and 1.5 mL of 2 × DMEM was mixed and added into the 24 well plate (500 µL/well), as the upper part. The 24 well plates were incubated at 37 °C for about 2 weeks.

### Plasmid Transfection

LentiCRISPRv2 was used to produce Cas9‐mediated PPDPF/SOS1 knockout lentivirus. PPDPF was cloned into pHAGE‐fEF1a‐IRES‐ZsGreen vector. To obtain stable cell lines, PDAC cell lines were transfected with lentivirus for 24 h, followed by puromycin treatment or GFP sorting. The sequences of PPDPF/SOS1 primers are provided in Table S4, Supporting Information.

### Immunoprecipitation

Protein immunoprecipitation was performed according to a previous report.^[^ ^39^ ^]^ Briefly, HEK293T cells were washed three times by ice‐cold PBS and lysed in IP lysis buffer with protease inhibitors for 30 min. Then supernatants were incubated with beads overnight. Next day, the beads were pelleted and washed by IP lysis buffer for 3 times. Finally, the sample was determined by SDS‐PAGE western blot.

### GST Protein Purification

GST‐RBD, PPDPF, and KRAS were purified by using BL21 according to a previous report.^[^ ^39^ ^]^ Briefly, gene sequences were subcloned into pGEX‐4T‐1 vector. Vectors were transformed into BL21 bacteria. The protein production with pGEX‐4T‐1 was incubated for 5 h at 30 °C with 1 m m IPTG. Cells were pelleted down and resuspended in PBS with protease inhibitor. They were sonicated for 40 min and Triton X‐100 was added to a final concentration of 1% for 30 min. This was followed by centrifuging at 12 000 × g for 20 min at 4 °C. The supernatant was incubated with GST beads for 1 h at 4 °C. The beads were pelleted down, washed for 5 times with PBS. Then the beads were incubated with Thrombin for 16 h at 4 °C. Bradford reagent was used to examine the concentration of the purified protein.

### 6xHis Protein Purification

SOS^H^, SOS^PH^, SOS^DH^, SOS^REM^, SOS^CDC25^, SOS^PR^, SOS^Cat^ (also named SOS1^Cat‐R1^, SOS1^Cat‐R2^ or SOS1^Cat‐R1/R2^ represented mutation among 807–814, 896–909 or 807–814/896–909 of SOS^Cat^, respectively; the site of mutation is showed in Table S2, Supporting Information) were cloned into the ProEX HTb vector.^[^ ^22b^ ^]^ Briefly, the vectors were transformed into BL21 (Rosta) with TB medium supplemented with Ampicillin. The BL21 with ProEX HTb vector was incubated for 16 h at 18 °C with 1 mm IPTG. Cells were collected by centrifugation for 20 min at 6000 × g, resuspended in buffer A and frozen at −80 °C. Protein production was sonicated for 40 min and Triton X‐100 was added to a final concentration of 1% for 30 min. Then cells were incubated with His beads for 1 h at 4 °C. The beads were pelleted down and washed for 4 times with buffer A. The protein productions were eluted by buffer B. Bradford reagent was used to examine the concentration of the purified protein.

### GEF Activity Assay

According to previous report,^[^ ^40^ ^]^ guanine nucleotide exchange assay uses fluorescent MANT‐GDP. Briefly, loading MANT‐GDP on KRAS was performed in low Mg^2+^ buffer. 100 µL KRAS was mixed with 100 µL of 2× MANT‐GDP loading buffer. Then the loading efficiency was measured on the fluorimeter. 14 µL of nucleotide exchange buffer or MANT‐GDP loaded KRAS was added into a 384‐well microplate. Before measuring the fluorescence, a working solution of GppNHp, SOS^Cat^ and/or PPDPF was prepared. The “Pause” button on the display was pressed to eject the plate and carry out 20 runs, then 1 µL GppNHp was added with SOS1^Cat^ and/or PPDPF to respective wells. The “Continue” button was pressed to insert the plate and continue reading fluorescence every 15 s at room temperature (25 °C) for at least 70 min. The fluorescence intensity was set at the first time point after the addition of either buffer or GppNHp to 1 and the relative fluorescence of each of the later time points was calculated.

### GTP Binding Assay

GTP binding assay was performed according to Pierce GTPase Enrichment Kit (ThermoscientifiC,88 314). A protein assay was performed to measure PPDPF or SOS1^Cat^ protein concentration. Then 1 µL of 0.5 m EDTA was added to each sample, mixed and incubated for 5 min at room temperature. Desthiobiotin‐GTP stock was added into each sample incubating for 10 min at room temperature with or without MgCl_2_, which was required GTP probe labeling. 8 m Urea/IP Lysis Buffer and High Capacity Streptavidin Agarose resin slurry was added to each sample, followed by incubating for 1 h at room temperature with constant mixing on a rotator. Samples were centrifuged at 1000 × g for 1 min to pellet resin. Supernatant was removed. 500 µL of 4 m Urea/IP Lysis Buffer and vortex were added briefly to mix. These steps were repeated two additional times. Eluted proteins were analyzed by SDS‐PAGE and Western blot.

### Microscale Thermophoresis (MST)

According to previous reports,^[^ ^41^ ^]^ MST was carried out on Monolith NT.115 instrument. 10 µm PPDPF in PBS with 10 mm MgCl_2_ (pH 7.3) was incubated with fluorochrome. The GppNHp (GTP) was diluted with 16 dilutability. Next, 10 µL PPDPF was mixed with 10 µL GppNHp. The mixed solution was added in siphon. The signal was detected by NT.115 instrument.

### Identification of GTP Binding Site by LC‐MS/MS Analysis

The purified PPDPF protein sample was added with 1 µL 0.2 m EDTA for 10 min. Then GppNHp and MgCl_2_ were added into solution for 20 min. The sample was determined by SDS‐PAGE. The target fragment was cut off for subsequent mass spectrometry experiments. After reduction and alkylation, trypsin (mass ratio 1:50) was added and incubated at 37 °C for 20 h. The sample was desalted and lyophilized. Then the protein was redissolved in 0.1% FA solution and stored at −20 °C. Solution A was an aqueous solution of 0.1% formic acid, and solution B was an aqueous solution of 0.1% formic acid in acetonitrile (84%). After the column was equilibrated with 95% solution A, the sample was loaded into trap column by automatic injector. The mass charge ratio of polypeptide and polypeptide fragments was collected by using LC‐MS/MS (nanoLC‐QE). The raw file of mass spectrometry was retrieved from the corresponding database by mascot2.2 software, and the results of protein identification were obtained.

### Prediction of PPDPF–SOS1 Interaction

Multiple online services (including trRefineRosetta,^[^ ^42^ ^]^ I‐TASSER,^[^ ^43^ ^]^ QUARK,^[^ ^44^ ^]^ and tfold [https://drug.ai.tencent.com/console/cn/protein]) were employed to predict the protein structure of PPDPF, and every output contained about 5 decoys. Masif site^[^ ^45^ ^]^ was use to predict the amino acids on protein surface involved in the protein complex. By observing the distribution of such amino acids on every decoy, a decoy of TrRefineRosetta was selected according to the experimental results.

In order to obtain the possible spatial structure of PPDPF‐SOS1 dimer, the SOS1 structure in 1NVU was chosen. The local docking process of the online service Haddock^[^ ^46^ ^]^ was used, and the candidate amino acids on contact surface were predicted by masif site. According to the sorting by haddock, the TOP 2 dimer decoys were selected. The adjacent amino acids of the two PDBs on the interface were calculated by COCOMAPS,^[^ ^23^ ^]^ and the possible key amino acids were predicated by pydockeneres.^[^ ^47^ ^]^

### Statistics

Survival curves were plotted by the Kaplan–Meier method and analyzed by the log‐rank test. Statistical analyses were performed by GraphPad Prism 5 and SPSS 22 (IBM) software. The results are representative of at least three independent experiments performed in triplicate and are expressed as the means ± SD. The data were analyzed using two‐tailed unpaired Student's *t*‐test. Correlation was analyzed by Pearson correlation analysis. The criterion for significance was *p* < 0.05 for all comparisons. Statistical analysis used in each panel was described in the figure legends.

## Conflict of Interest

The authors declare no conflict of interest.

## Author Contributions

The author contribution is as follows: study design and conduct (Q.N., D.X., J.L.); data collection (Q.N., B.Z., Y.J., Q.Z., X.L., N.M., Y.S.); data analysis and interpretation (Q.N., B.Z., H.J., F.Z., Y.W., S.X., E.Z., Y.Y., T.C.,F.Y., H.C., J.H., J.X., X.D., X.Q., K.D., C.S., W.Z., M.W., K.W., R.L., L.Q., W.C., Z.L.); drafting paper (Q.N.); revising paper content and approving the final version of paper(S.C., X.W., D.X.J.L.).

## Supporting information

Supporting Information

## Data Availability

The data that support the findings of this study are available from the corresponding author upon reasonable request.
