# Supplementary material for: PPDPF Promotes the Development of Mutant KRAS‐Driven Pancreatic Ductal Adenocarcinoma by Regulating the GEF Activity of SOS1
Source: Adv Sci (Weinh). 2022 Dec 1;10(2):2202448. doi: 10.1002/advs.202202448 (PMC9839844; doi:10.1002/advs.202202448)
Supplement: Supplementary file 1 — Supporting Information [file ADVS-10-2202448-s001.pdf]

ADVANCED SCIENCE
Open Access

Supporting Information

for *Adv. Sci.*, DOI 10.1002/advs.202202448

PPDPF Promotes the Development of Mutant KRAS-Driven Pancreatic Ductal Adenocarcinoma by Regulating the GEF Activity of SOS1

Qian-Zhi Ni, Bing Zhu, Yan Ji, Qian-Wen Zheng, Xin Liang, Ning Ma, Hao Jiang, Feng-Kun Zhang, Yu-Rong Shang, Yi-Kang Wang, Sheng Xu, Er-Bin Zhang, Yan-Mei Yuan, Tian-Wei Chen, Fen-Fen Yin, Hui-Jun Cao, Jing-Yi Huang, Ji Xia, Xu-Fen Ding, Xiao-Song Qiu, Kai Ding, Chao Song, Wen-Tao Zhou, Meng Wu, Kang Wang, Rui Lui, Qiu Lin, Wei Chen, Zhi-Gang Li, Shu-Qun Cheng, Xiao-Fan Wang, Dong Xie and Jing-Jing Li**

Supporting Information

PPDPF promotes the development of mutant KRAS -driven pancreatic ductal adenocarcinoma by regulating the GEF activity of SOS1

Qian-Zhi Ni, Bing Zhu, Yan Ji, Qian-Wen Zheng, Xin Liang, Ning Ma, Hao Jiang, Feng-Kun Zhang, Yurong Shang,Yi-Kang Wang, Sheng Xu, Er-Bin Zhang, Yan-Mei Yuan, Tian-Wei Chen, Fen-fen Yin, Hui-Jun Cao, Jing-Yi Huang, Ji Xia, Xu-Fen Ding, Xiao-Song Qiu, Kai Ding, Chao Song, Wen-Tao Zhou, Meng Wu, Kang Wang, Liu Rui, Qiu Lin, Wei Chen, Zhi-Gang Li, Shu-Qun Cheng, Xiao-fan Wang, Dong Xie* and Jing-Jing Li *

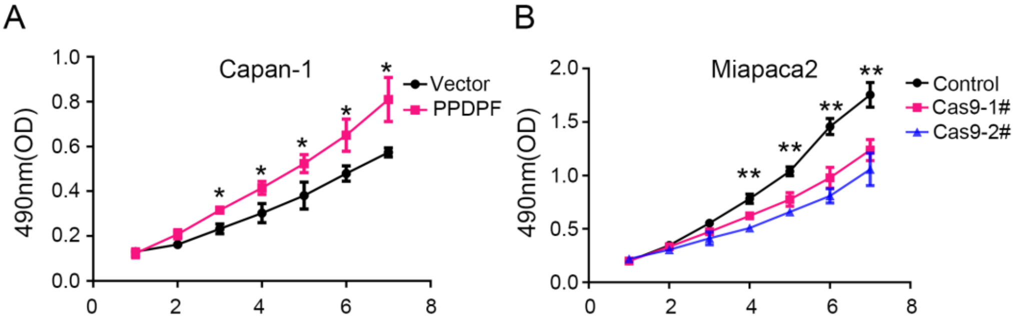

Supplement figure 1. PPDPF promotes the growth of pancreatic cancer cells. A, B, The effects of PPDPF overexpression (A) or knockout (B) on the proliferation of pancreatic cancer cells are evaluated by MTT assay. Data were analyzed with two-tailed unpaired Student's t test and expressed as mean±SD. $*p < 0.05$; $**p < 0.01$.

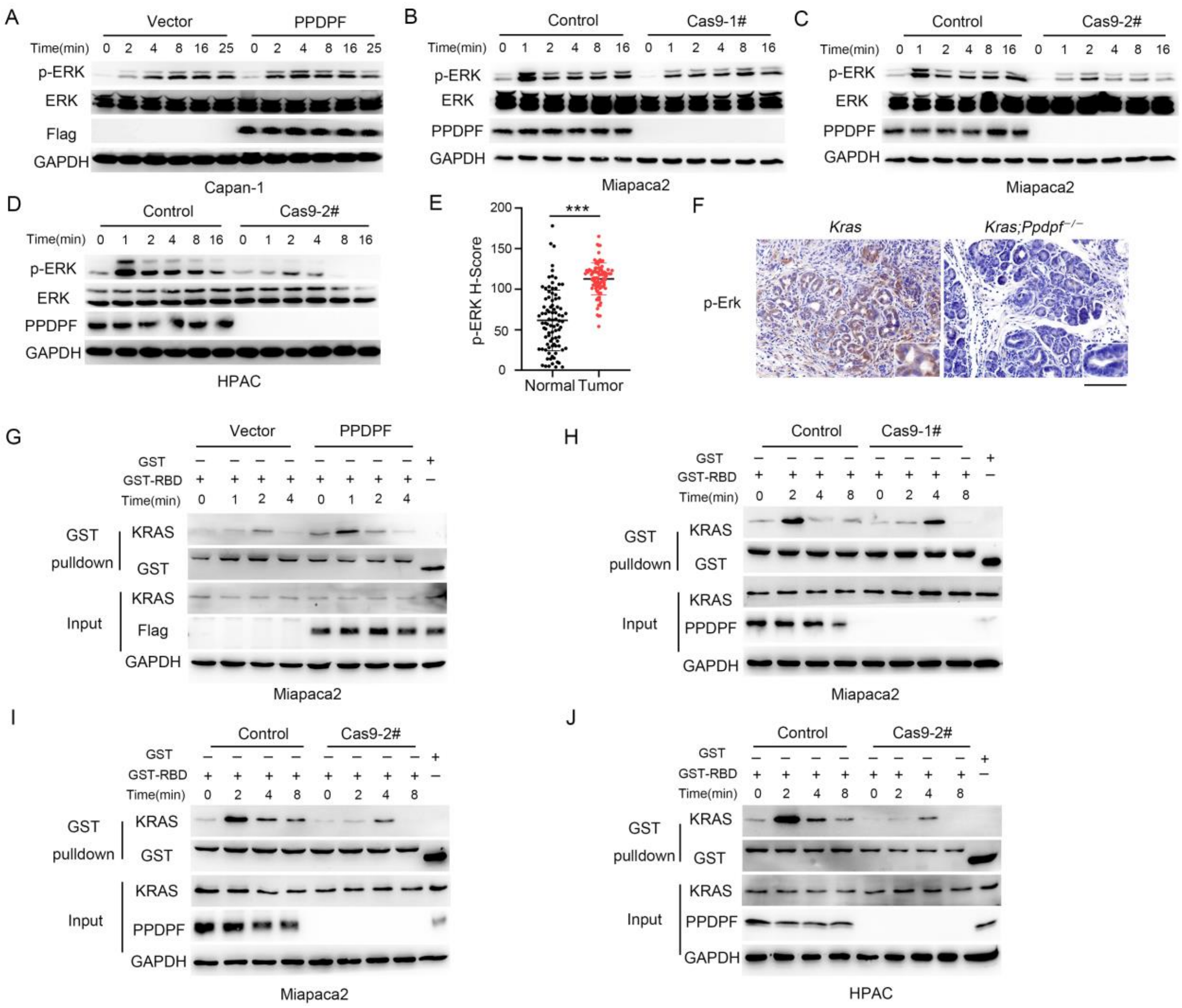

Supplement figure 2. PPDPF activates MAPK signaling in PDAC. A, Western blot analysis of p-ERK in control and PPDPF-overexpressing Capan-1 cells with EGF treatment. B, C, D, Western blot analysis of p-ERK in control and PPDPF knockout Miapaca2 and HPAC cells with EGF treatment. E, H-scores of p- ERK protein in PDAC tissues (T, n=90) and the matched adjacent noncancerous tissues (N, n=90) in the tissue microarray ($p < 0.0001$). F, Immunohistochemical staining for p-Erk in the pancreases from Pdx1-Cre; KRAS^{G12D} (*Kras*) or Pdx1-Cre; Kras^{G12D};Ppdpf$^{f/f}$ (*Kras*;*Ppd*pf$^{-/-}$) mice; Scale bar, 100μm. G, The level of KRAS-GTP in control and PPDPF-overexpressing cells with EGF treatment was detected by GST- RBD pulldown assay. H, I, J, The level of KRAS-GTP in control and PPDPF-knockout PDAC cells with EGF treatment was examined by GST-RBD pulldown assay. Data were analyzed with two-tailed unpaired

Student's t test. Data were expressed as mean±SD. ***$p < 0.001$.

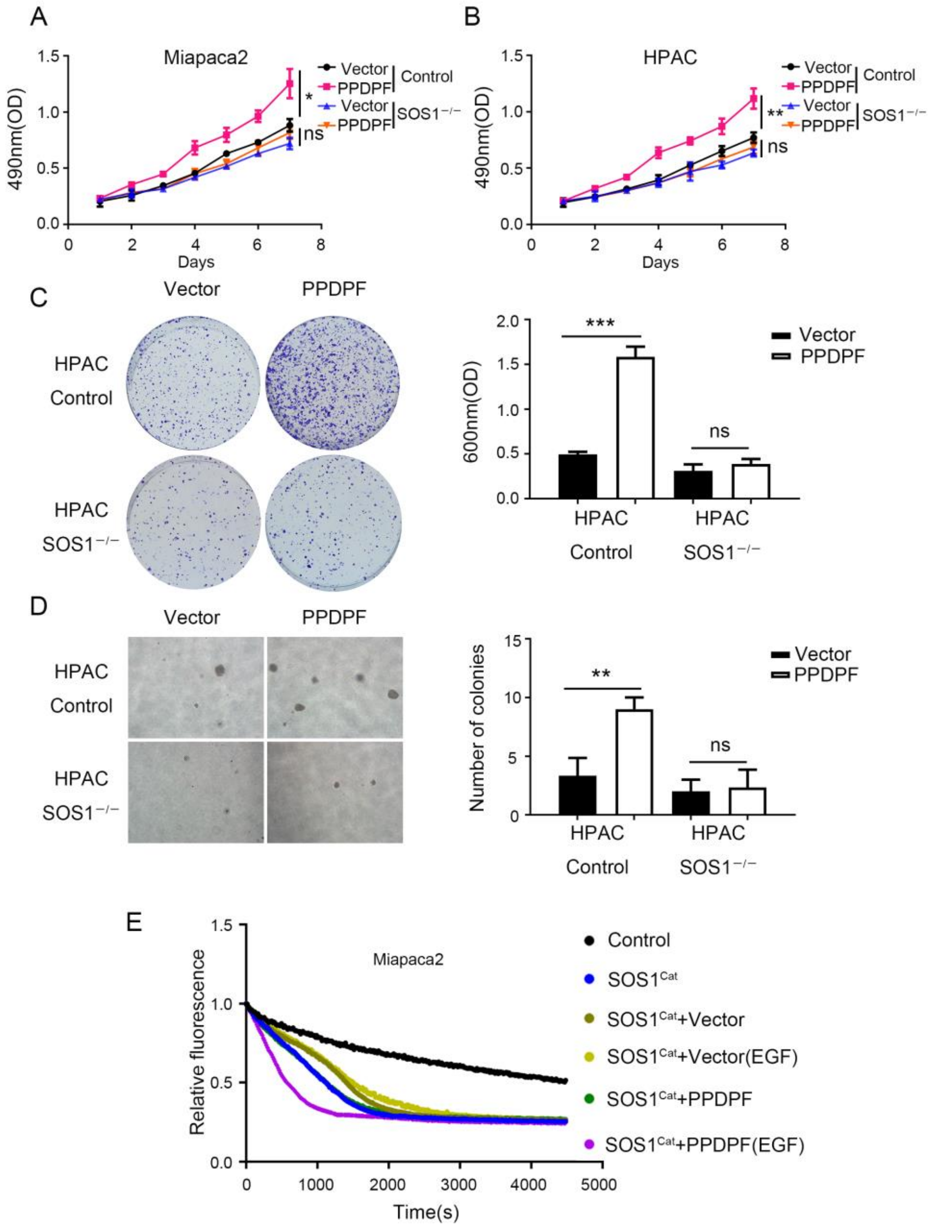

Supplement figure 3, The tumor-promoting effect of PPDPF depends on the GEF activity of SOS1. A, The growth of control and PPDPF-overexpression Miapaca2 cells in the presence or absence of SOS1 was detected by MTT assay. B, C, D, The growth of control and PPDPF-overexpressing HPAC cells in the presence or absence of SOS1 was detected by MTT assay (B), Crystal violet assay (C) and Soft agar assay (D). E, The GEF activity of $SOS1^{Cat}$ with or without PPDPF (EGF) from Capan-1 cells. Data were analyzed with two-tailed unpaired Student's t test. Data were

expressed as mean±SD. *p < 0.05; **p < 0.01; ***p < 0.001, ns: not significant.

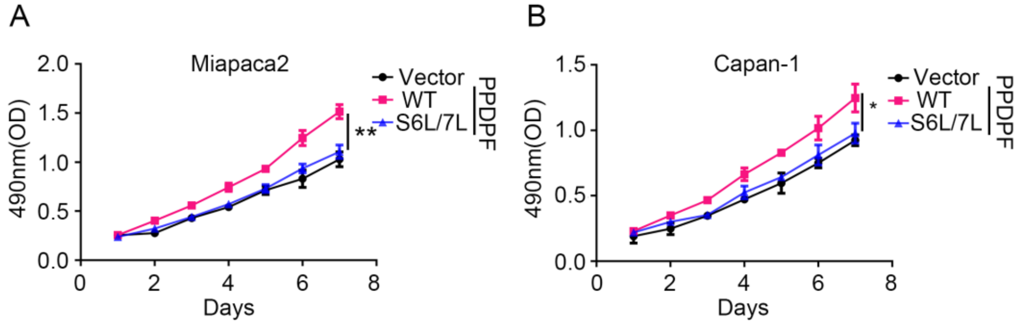

Supplement figure 4, GTP-binding ability is required for the tumor-promoting function of PPDPF. A, B, MTT assay was used to determine the proliferation ability of the indicated PDAC cells. Data were analyzed with two-tailed unpaired Student's t test. Results were expressed as mean±SD. *p < 0.05; **p < 0.01.

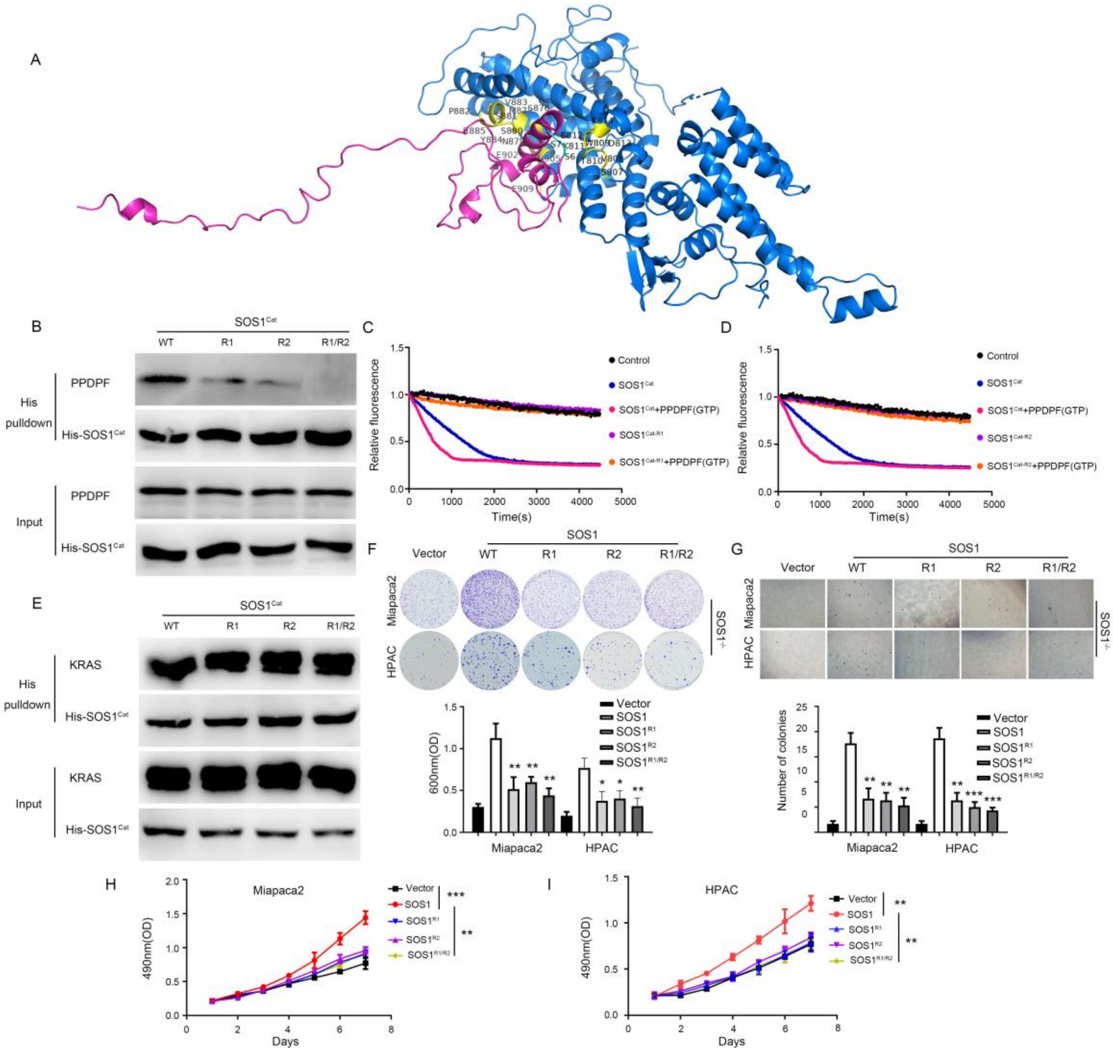

Supplement figure 5, GTP transfer from PPDPF to SOS1 is indispensable for the tumor-promoting effect of PPDPF-SOS1 axis. A, The whole picture of PPDPF-SOS1 interaction predicted by an integrated analysis. B, Interactions between PPDPF and WT SOS1^{Cat}, and the indicated SOS1^{Cat} mutants *in vitro*. C, D, The GEF activity of SOS1^{Cat-R1} (C) or SOS1^{Cat-R2} (D) with or without PPDPF-GTP. E, Interactions between KRAS and SOS1^{Cat}, SOS1$^{Cat-R1,}$ SOS1^{Cat-R2} and SOS1$^{Cat-R1/R2}$ *in vitro*. F, G, H, I, Crystal violet assay, soft agar assay and MTT assay were employed to determine the colony formation ability and growth of control, SOS1 WT and SOS1 mutants-overexpressing PDAC cells. Data were analyzed with two-tailed unpaired Student's t test. Results were expressed as mean±SD. $*p < 0.05$; $**p < 0.01$;$***p < 0.001$.

Supplemental Table 1 Correlation between PPDPF expression and clinicopathological characteristics of PDAC patients

	PPDPF Expression			
	Total	High	Low	
Characteristic	N=90	N=45	N=45	p-Value
Gender				0.192
Male	56	25	31	
female	32	20	15	
Age.years				0.809
> 60	66	34	32	
≤60	24	11	13	
Tumor size				0.031*
≤2cm	16	4	12	
2-5cm	65	34	31	
> 5cm	9	7	2	
Lymph node metastasis				0.829
Negative	88	44	44	
Positive	2	1	1	
Distant metastasis				1
Negative	88	44	44	
Positive	2	1	1	
TNM Stage				0.018*
I	18	5	13	
II	59	29	30	
III	9	8	1	
IV	4	3	1	
Histological grade				0.038*
I	0	0	0	
II	27	9	18	
III	63	36	27	

Supplemental Table 2 The critical amino acids within the two regions of SOS1

PPDPF	PPDPF_index	SOS1	SOS1_index	ele_rec	vdw_rec	ele_lig	vdw_lig
SER	6	TRP	809	-1.42905	-4.02449	-0.27409	-3.68468
SER	6	ASP	813	-1.42905	-4.02449	-0.40058	-0.19598
SER	6	GLU	812	-1.42905	-4.02449	-3.70308	-0.82812
SER	6	SER	807	-1.42905	-4.02449	-0.01049	-0.32765
SER	6	LYS	811	-1.42905	-4.02449	0.77482	-3.91585
SER	6	VAL	808	-1.42905	-4.02449	-0.05109	-0.23016
SER	6	THR	810	-1.42905	-4.02449	0.15089	-3.86641
SER	6	LYS	814	-1.42905	-4.02449	3.16993	-1.67297
SER	6	SER	880	-0.48484	-3.00341	0.33145	-3.08565
SER	6	ASN	879	-0.48484	-3.00341	-1.60718	-5.36885
SER	6	VAL	883	-0.48484	-3.00341	0.10249	-0.25981
SER	6	MET	878	-0.48484	-3.00341	0.06252	-0.43777
SER	6	SER	876	-0.48484	-3.00341	-0.91903	-5.90889
SER	6	SER	881	-0.48484	-3.00341	0.53349	-1.81641
SER	6	ARG	885	-0.48484	-3.00341	0.61472	-0.55425
SER	6	TYR	884	-0.48484	-3.00341	0.23709	-5.06195
SER	6	PRO	882	-0.48484	-3.00341	0.04746	-0.57185
SER	7	LYS	811	-1.59965	-0.36575	0.77482	-3.91585
SER	7	GLU	812	-1.59965	-0.36575	-3.70308	-0.82812
SER	7	ASP	813	-1.59965	-0.36575	-0.40058	-0.19598
SER	7	THR	810	-1.59965	-0.36575	0.15089	-3.86641
SER	7	LYS	814	-1.59965	-0.36575	3.16993	-1.67297
SER	7	ASN	879	0.37161	-2.67677	-1.60718	-5.36885
SER	7	TYR	884	0.37161	-2.67677	0.23709	-5.06195
SER	7	HIS	905	0.37161	-2.67677	-0.95334	-3.89753
SER	7	GLU	902	0.37161	-2.67677	-0.11206	-0.21187
SER	7	GLU	909	0.37161	-2.67677	-1.15557	-2.76172
SER	7	ARG	885	0.37161	-2.67677	0.61472	-0.55425
SER	7	SER	880	0.37161	-2.67677	0.33145	-3.08565

Supplemental Table 3 Primers for mouse genotyping

Gene	Forward	Reverse
mPpdpf	GGCCTTACTCACTTGTACTGCTGTC	CCATCGATcGGACGGGGGCCCAGCGCTGGCTGTG
mKras	CTAGCCACCATGGCTTGAGT	TCCGAATTCAGTGACTACAGATG
Pdx1-Cre	AGGTGTAGAGAAGGCACTCAGC	CTAATCGCCATCTTCCAGCAGG
mP53	CACAAAAACAGGTTAAACCC	AGCACATAGGAGGCAGAGAC

Supplemental Table 4 Primers for plasmid construction and RT-qPCR

Gene	Forward	Reverse
PPDPF	CGGGGTACCATGGCGGCCATCCCCTCCAGCGGCT	CCATCGATcGGACGGGGGCCCAGCGCTGGCTGTG
PPDPF-CAS9-1#	CACCGATGGCGGCCATCCCCTCCAG	AAACCTGGAGGGGATGGCCGCCATC
PPDPF-CAS9-2#	CACCGCAGCAGTACCGAGTGCCCCG	AAACCGGGGCACTCGGTACTGCTGC
SOS1-CAS9-1#	CACCGGAGAACGCGCCCAAGTGGCG	AAACCGCCACTTGGGCGCGTTCTCC
SOS1	TGCTCTAGAATGCAGGCGCAGCAGCTGCCCTACG	CCATCGATCGGAAGAATGGGCATTCTCCAACAGT
$SOS1^{CAT}$	TGCTCTAGAATGCAGATGAGGCTGCCTAGTGCTGATG	CCATCGATcACCTGGTCTTGGGTTTGATGGACGA
$SOS1^{REM}$	TGCTCTAGAATGGAA AGGATGCTTGATGTA ACAATGC	CCATCGATcACCTGGTCCATTGTCTCTTGCAATT
$SOS1^{CDC25}$	TGCTCTAGA ATGCATAATATTACATTTCAGAGTTCACCTCCCACA	CCATCGATcACCTGGTCTTGGGTTTGATGGACGA
$SOS1^{H}$	TGCTCTAGAATGCAGGCGCAGCAGCTGCCCTACG	CCATCGATcTCCTGAGGTGGAAGGCTCTTCGTCA
$SOS1^{PH}$	TGCTCTAGA ATGGCATGTCGGTTTTATAGTCAGCAAA	CCATCGATcCAGTGTACTCCGGTACTGTAAAGAT
$SOS1^{DH}$	TGCTCTAGAATGGAACAAACTTACTATGATTTGGTAAAAGCATT	CCATCGATcAGATTCACTCAGTCTTCGTTTTGCA
$SOS1^{PR}$	TGCTCTAGA ATGAGGCATCCCACACCTCTGCAGC	CCATCGATCGGAAGAATGGGCATTCTCCAACAGT
KRAS	TCCCCCGGGCATGACTGAATATAAACTTGTGGTAG	ATAAGAATGCGGCCGC CATAATTACACACTTTGTCTTTGAC
RT-homo-PPDPF	GCGGTCTTCTCTGCAAATGGG	TGCTTTAGCTTGCTGGCTGGT
